# Molecular Marker Study of Particulate Organic Matter in Southern Ontario Air

**DOI:** 10.1155/2017/3504274

**Published:** 2017-09-17

**Authors:** Satoshi Irei, Jacek Stupak, Xueping Gong, Tak-Wai Chan, Michelle Cox, Robert McLaren, Jochen Rudolph

**Affiliations:** ^1^Centre for Atmospheric Chemistry and Department of Chemistry, York University, 4700 Keele St., Toronto, ON, Canada M3J 1P3; ^2^Climate Chemistry Measurements and Research, Climate Research Division, Environment and Climate Change Canada, 4905 Dufferin Street, Toronto, ON, Canada M3H 5T4

## Abstract

To study the origins of airborne particulate organic matter in southern Ontario, molecular marker concentrations were studied at Hamilton, Simcoe, and York Gateway Tunnel, representing industrial, rural, and heavy traffic sites, respectively. Airborne particulate matter smaller than 10 *μ*m in aerodynamic diameter was collected on quartz filters, and the collected samples were analyzed for total carbons, 5-6 ring PAHs, hopanes,* n*-alkanes (C_20_ to C_34_), and oxygenated aromatic compounds. Results showed that PAH concentrations at all three sites were highly correlated, indicating vehicular emissions as the major source. Meanwhile, in the scatter plots of *α*,*β*-hopane and trisnorhopane, concentrations displayed different trends for Hamilton and Simcoe. The slopes of the linear regressions for Hamilton and the tunnel were statistically the same, while the slope for Simcoe was significantly different from those. Comparison with literature values revealed that the trend observed at Simcoe was explained by the influence from coal combustion. We also found that the majority of oxygenated aromatic compounds at both sites were in the similar level, possibly implying secondary products contained in the southern Ontario air. Regardless of some discrepancies, absolute principal component analysis applied to the datasets could reproduce those findings.

## 1. Introduction

Studying chemical composition of airborne particulate matter (PM) is very important to understand the terrestrial radiative forcing [1] and the adverse health effect [2]. Organic fraction in airborne particulate matter (particulate organic matter (POM)) is potentially associated with these issues. Numerous studies have demonstrated that there is no doubt that the POM is the major constituent of airborne particulate matter (e.g., [3]). To date, a number of molecular marker studies have been done for source identification and apportionment of POM [4–8].

The objective here is to better understand the origins of airborne POM during a case study in southern Ontario, Canada. A field campaign was carried out in the summer of 2000 at two locations in southern Ontario, Hamilton, and Simcoe. During the study, hourly averaged mixing ratios of SO_2_ and 24-hour averaged molecular marker and total carbon (TC) concentrations in PM were measured. To understand the influence of vehicular emissions, which were expected to be one of the major sources of POM, four times of tunnel studies were carried out in 2000 and 2001. Obtained concentrations were compared to investigate their relationships, which give clues for the source identification. Wind sector dependency was also evaluated to identify the directions of emission sources, and absolute principal component analysis (APCA) was applied to get deeper insight into the sources identification and apportionment.

## 2. Methodology

### 2.1. Field Study

**Figure 1:**
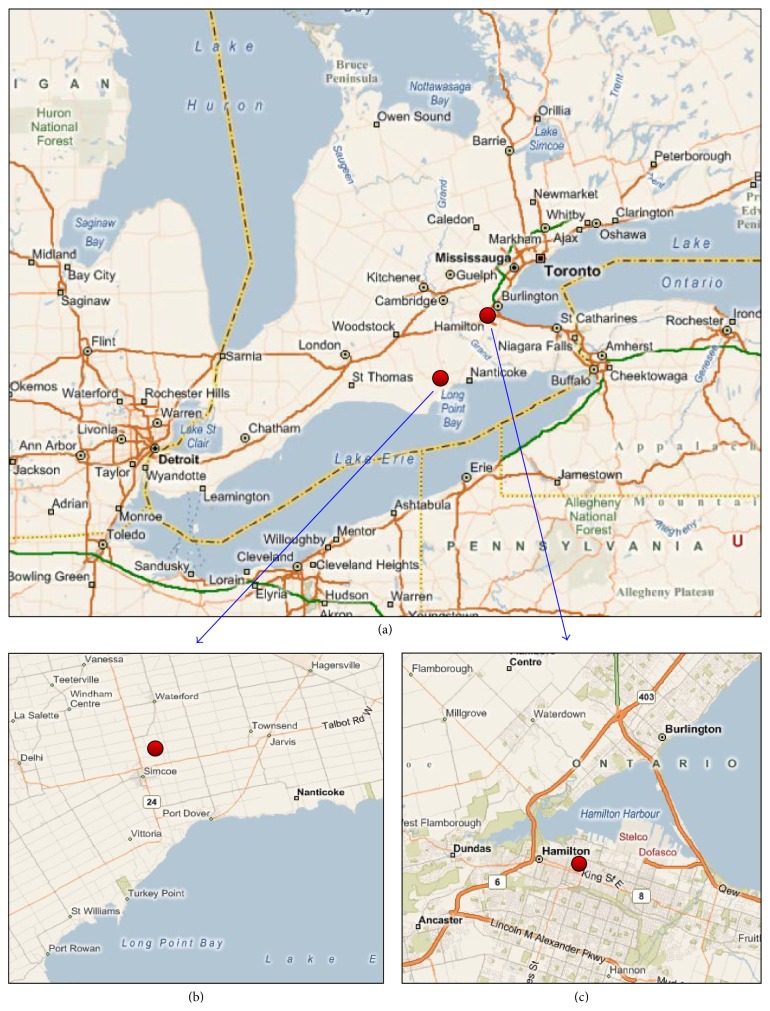
Location of sampling stations for the SONTAS 2000: southern Ontario (a), Simcoe (b), and Hamilton (c).* Source*: http://mappoint.msn.com.

Southern Ontario Aerosol Studies (SONTAS 2000) were carried out from June 23 to July 19, 2000, at a measurement station located in downtown Hamilton (43° 15′ N, 79° 51′ W) and another site located near the agricultural field of University of Guelph in the town of Simcoe (42° 51′ N, 80° 16′ W) (Figure 1). These sites represent an industrial site and a rural site in southern Ontario, respectively. During the study, airborne particulate matter smaller than 10 *μ*m in aerodynamic diameter (PM_10_) was collected on 8 × 10-inch Pallflex Tissuquartz filters (Pall Corp., NY, USA) using high volume air samplers equipped with PM_10_ separators (Tisch Environmental Inc., Cleves, OH, USA). The filters were heated to 750°C for more than 4 h prior to the use. The sampling flow rate was set to 1.13 m^3^ min^−1^, compatible with the sampling requirement for PM_10_ collection. Sampling duration was 24 h during the campaign, except some periods: 4 h sampling was made on July 1st and 2nd at Hamilton and Simcoe, and 12 h sampling was made on July 13 at Simcoe. Samples on July 14 and 15 at Hamilton and July 18 at Simcoe were not collected. In total, 28 and 33 PM_10_ samples and 7 and 10 field blanks were collected at Hamilton and Simcoe, respectively. It should be noted that we refer to the date on which the sampling started as the sample name. It should also be noted that the 4 h samples collected on July 1st and 2nd were excluded from the analysis because we found that the impact of blank values on the samples with small loading was relatively high. In addition to the SONTAS, four times of tunnel studies were carried out at the York Gateway Tunnel (YGT) in downtown Toronto (June 1st, August 24th, and September 21st, 2000, and February 21st, 2001) to characterize molecular marker concentrations from vehicular emissions. Due to high molecular marker concentrations, only 4 h sampling was made. In total, five PM_10_ samples and one field blank were collected during the tunnel study. Each filter sample was cut into eight identical sizes, and then one or two segments of each sample were then used for the molecular marker and TC analysis.

### 2.2. Analysis

Here we analyzed the filter samples for the following nonpolar substances to trace primary POM: polycyclic aromatic hydrocarbons (PAHs) of benzo[a]pyrene (BaP), dibenz[a,h]anthracene (Db), indeno[1,2,3-cd]pyrene (Ind), benzo[ghi]perylene (BgP), and coronene (Cor) (purities > 99%, Sigma-Aldrich Canada, Oakville, ON, Canada); hopane series of trisnorhopane (TrisHp), norneohopane (NorHp), *α*,*β*-hopane (abHp), and *β*,*α*-hopane (baHp) (purities > 99%, Chiron AS, Norway); and *n*-alkanes with the carbon numbers from C_20_ to C_34_ (purities > 99%, Sigma-Aldrich Canada). The details of the nonpolar molecular marker analysis are described elsewhere [21]. Briefly, one or two pieces of each filter sample, which 40 and 200 ng of internal standards (benzo[a]pyrene-d_2_ and tetracosane-d_50_, Sigma-Aldrich Canada) were spiked onto, were extracted with 100 mL of PRA grade dichloromethane (Sigma-Aldrich Canada) using Soxhlet apparatus (Sigma-Aldrich Canada) for 16 hours. The extract was transferred to a 200 mL round bottom flask, and its volume was reduced to a few mL using a rotary evaporator (Buchi, New Castle, DE, USA). The concentrated extract and rinse from the flask were combined and filtrated using a 25 mm o.d. PTFE syringe filter with a pore size of 0.45 *μ*m (Chromatographic Specialties, Inc., Brockville, ON, Canada). The volume of extract was further reduced to near dryness in a 5 mL Reacti-Vial (Pierce, IL, USA) under a gentle stream of pure nitrogen (Praxair Canada, Mississauga, ON, Canada) and then dissolved in ~0.2 mL of hexane : benzene (2 : 1) mixture. Finally, 100 ng of triacontane-d_62_ (Sigma-Aldrich Canada), the third internal standard, was spiked to the concentrated extract. 5 *μ*l of the extract was directly analyzed by a GC-MS (HP 5890 and 5972, Agilent Technologies, USA) using two-minute splitless injection mode. Scanning and selected ion monitoring (SIM) were used to identify and quantify the molecular markers. The identification of the molecular markers was made by comparison with the retention time and the reference mass spectrum obtained by the analysis of chemical standards and from the NIST 98 mass spectrum library. For the purpose of quality control of GC-MS measurements, a reference standard mixture containing 100 ng mL^−1^ of each substance referred to above was measured after an injection of a sample extract.

We also analyzed the following 11 oxygenated aromatic compounds of polar substances: 4-nitrophenol (4-NO_2_-Phen), 2-hydroxybenzoic acid (2-OH-BA), 3-hydroxybenzoic acid (3-OH-BA), 4-hydroxybenzoic acid (4-OH-BA), ortho-phthalic acid (*o*-PhA), meta-phthalic acid (*m*-PhA), para-phthalic acid (*p*-PhA), 4-methylphthalic acid (4-Me-PhA), 1,2,3-benzenetricarboxylic acid (1,2,3-BA), 1,2,4-benzenetricarboxylic acid (1,2,4-BA), and 1,2,4,5-benzenetetracarboxylic acid (1,2,4,5-BA) (Sigma-Aldrich Canada). Analysis of these polar substances is described elsewhere [22]. Briefly, one or two pieces of each filter sample were spiked with* m*-toluic acid as an internal standard, and the filter sample was then extracted with a mixture of 10 mL of diethyl ether (Sigma-Aldrich Canada) and 1 mL of 0.1 M hydrochloric acid solution by stirring for 1.5 hours. In the strongly acidified water, the polar substances are partitioned to diethyl ether phase. The diethyl ether extract was filtered using the syringe filter referred to above. This extraction procedure was repeated two more times with 2 mL of the solvent mixture. The extracts were combined together, and the combined volume was reduced under a gentle flow of nitrogen until being dried. The dried extract was then dissolved in 100 *μ*l of 2 mM boric acid, the pH of which was preadjusted to 9.9 with sodium hydroxide. The extract prepared in this manner was analyzed by a capillary electrophoresis coupled with an UV/visible diode array detector (3DCE, Agilent Technologies).

The details of TC measurement are described elsewhere [23]. Briefly, a disc with 1.5 cm diameter was punched out from a segment of each filter sample and analyzed by a custom-made thermal desorption TC analyzer. The TC analyzer consists of a desorbing oven operated at 800°C and followed by an oxidation furnace and a flame ionization detector. The instrument quantifies CO_2_ evolving from the combustion of organic carbon and from thermal decomposition of inorganic carbonate. It should be noted that all concentrations of molecular markers and TC hereafter are blank-corrected.

Sulfur dioxide (SO_2_) was also measured at Hamilton and Simcoe. The ambient air was sampled from the rooftop and the measurements were made using SO_2_ analyzers (Model 43C, Thermo Environmental). Hourly meteorological elements for Hamilton were obtained from the meteorological archives in Environment Canada. For Simcoe, hourly wind speeds and sectors were provided by Rotek Environmental Inc. (Hamilton, ON, Canada).

### 2.3. APCA

APCA has been applied to our POM dataset to attempt better understanding of source types. The details of APCA have been described elsewhere [24]. The minimum number of components has been determined by scree plots, and the optimum number of components was then determined by checking scree plots and distribution patterns of molecular markers in each component.

## 3. Results and Discussion

### 3.1. Observation Records

The following are important records written at the site of Simcoe; grass of the campus yard around the sampling site was mowed on June 26–28 and July 19; smells of burning were recorded at Simcoe between July 8 and July 14 from west of the sampling site: clear influence of a plume from the Nanticoke coal-fired power plant (located approximately 20 km southeast of the sampling site of Simcoe) was visually observed at Simcoe on July 12. This event may have influenced the molecular marker concentrations of the sample, namely, July 11 (i.e., the sample collected from July 11 through July 12).

### 3.2. Concentration Level

The mean mixing ratios ± standard deviations (SD) of SO_2_ at Hamilton and Simcoe were 3.9 ± 5.9 and 2.6 ± 1.5, respectively. The minimum and maximum mixing ratios for these sites were 0–30 ppbv and 0–12 ppbv, respectively. Although the difference between the mean concentrations is insignificant, the comparison of variations indicates local emission source(s) of SO_2_ at Hamilton. Observed mean concentrations and concentration ranges of TC and molecular markers during our field studies are summarized in Table 1. Overall, there were differences between Hamilton and Simcoe in the concentrations of TC, PAHs, hopanes, some* n*-alkanes, and oxygenated aromatic compounds such as 3-OH-BA and 4-Me-PhA. The differences indicate the air quality difference between the industrial urban site and the rural site in southern Ontario. There were also substances that did not show the air quality difference, such as TrisHp, the majority of *n*-alkanes, and oxygenated aromatic substances. This implies that for these substances there were some major other local emission sources at Simcoe or that those substances were contained in the large air mass covering southern Ontario. The mean concentrations of these molecular markers at the YGT were much higher than those at Hamilton (3 to 20 times higher mean concentrations).

Comparison of the mean marker concentrations with those observed in different field studies is shown in Table 2. The mean PAH concentrations observed at Hamilton during SONTAS 2000 were compared with the concentrations observed at the same sampling station in 1990 [9], and we found that the concentration ranges during SONTAS 2000 were more than 10 times lower than those reported in the literature. This is probably explained by the fact that the factory emissions, which were the major POM source in Hamilton, may have been reduced substantially. Comparison with the other studies at other locations reveals that the mean concentrations of molecular markers during SONTAS 2000 were almost of the same level as the concentrations observed in other cities (Philadelphia, Atlanta, and Houston) of the United States [10–12]. It is also interesting to compare the concentrations observed during our tunnel studies with the other tunnel studies in Los Angeles [13]. The variation patterns of molecular markers from our tunnel studies and their tunnel studies agreed (Table 2), suggesting that these molecular markers can be used as the fingerprint for vehicular emission overall.

### 3.3. Time Series Variation

Time series plot of daily frequency of wet deposition (rain and fog) occurrence at Hamilton and time series plots of TC and molecular marker concentrations at both Hamilton and Simcoe are shown in Figures  S-1 and S-2 in Supplementary Material available online at https://doi.org/10.1155/2017/3504274, respectively. The daily frequency of wet deposition occurrence is the counts of hourly rain and fog events relative to the sampling hour (i.e., 24 h or 12 h). Comparison of these plots shows that the frequency of wet deposition was not negatively correlated with the concentrations of any species (*r*^2^ < 0.19). However, the poor correlations do not necessarily rule out the washout of POM by the precipitations because the molecular marker concentrations depend on the combination of many factors, such as wind speed, strength of emission rate, and frequency and strength of wet deposition. The wet depositions were observed at both sites in the similar days, and this can be explained by the fact that the precipitations observed at Hamilton and Simcoe were the regional scale events.

Time series plot of hourly averaged SO_2_ mixing ratios at Hamilton showed sharp rises and falls, each of which lasted for 1 to 10 hours (Figure  S-2a). These observations cannot be explained by diesel emissions only because the mobile sources would not continuously contribute to the input of SO_2_ into Hamilton's air. The observations are more likely explained by the combination of diesel emissions and factory emissions combusting coal or cokes. Time series plot of hourly averaged SO_2_ mixing ratios at Simcoe shows much smaller variations due to considerably lower mixing ratios than Hamilton (Figure  S-2b). Nevertheless, some small peaks were observed. These peaks suggest that there are local sources of SO_2_ at Simcoe.

Time series variation of TC concentrations showed marginal variations at both sites and any remarkably high or low concentration episode was not observed (Figures  S-3a and 3b). The variations were not as large as the variations of molecular markers and SO_2_, indicating that there are other carbonaceous substances in the filter samples.

In the time series plot of PAHs at Hamilton, the highest concentrations of the PAHs were observed on June 23, and this was a local event because such a high concentration was not observed at Simcoe (Figures  S-3c and 3d). Marginally high concentrations of PAHs were also observed from July 3–7 and July 10–13, and the concentrations were low between these episodes, during which precipitation was recorded. Those levels of concentrations were more likely usual at Hamilton. At Simcoe, the levels of PAHs were significantly lower than those at Hamilton; however, there were still local high concentration episodes observed: July 11 and 13. As mentioned earlier in our records, the episodes were likely the influence of the emissions from the Nanticoke power plant located near the site.

Time series plot of hopanes at Hamilton showed similar variation to those of PAHs (Figures  S-3e and 3f). abHp, an indicator for vehicular emissions [13, 25], was the highest level of the hopane series; therefore, it is likely that the major source of PAHs and hopanes was vehicular emissions. The variations of hopanes at Simcoe seem to be different from those of PAHs. Different variations and marginal magnitude of abHp concentrations suggest that their major source may be different from the vehicular emissions.

Time series plots of the sum of all* n*-alkane (C_20_ to C_34_) concentrations and its carbon preference index (CPI) demonstrate the variable influence of* n*-alkane from anthropogenic and natural emissions (Figures  S-3g and 3l). CPI is defined as follows [26, 27]:

(1) $$\begin{array}{c} CPI=\frac{1}{2}\left[\;\left(\;\frac{\sum_{i}^{z}odd}{\sum_{i}^{z-1}even}\;\right)+\left(\;\frac{\sum_{i}^{z}odd}{\sum_{i+1}^{z+1}even}\;\right)\;\right], \end{array}$$

where ∑odd, ∑even,* i*, and *z* stand for the sum of odd carbon number* n*-alkane concentrations, the sum of even carbon number* n*-alkane concentrations, and the start and end of carbon number range to be summed, respectively. CPI is an indicator to see if* n*-alkanes are originated from petroleum-related emissions (CPI = ~1) or plant wax origin (CPI > 3) [28–31]. It has been also reported that low CPI was also found in biomass burning [32–34].

The sum of all* n*-alkane concentrations at Hamilton showed remarkably high concentrations on July 5 and 10 with the CPIs < 2 (Figure  S-3g). This is evidence that there was strong influence from the anthropogenic emissions on those samples. If these samples were influenced by biomass burning (such as agricultural waste burning), we expect that the influence would be found in baHp, which is relatively a fresh hopane and can be an indicator of biomass burning [32]. However, such impact was not observed in baHp concentrations. The observations are, therefore, clearly explained by the input from the anthropogenic emissions. The samples collected on June 24, 25, 26, and 30 and July 8 at Hamilton showed CPIs > 3, an indication of the majority of plant wax* n*-alkanes. However, the magnitudes of the concentrations were smaller than those observed in the other days, suggesting minor contribution of plant wax* n*-alkanes.

The variation of* n*-alkane concentrations at Simcoe was different from that at Hamilton (Figure  S-3h). A high concentration episode can be found on July 13 with CPI < 2. The observation is consistent with the indications found in the PAH and hopane molecular markers that there was a remarkably high influence from the anthropogenic emissions. Besides this apparent episode, many of the samples at Simcoe showed low concentrations with high CPIs. Particularly, the observations in the period of June 26–28 were consistent with our records that the yard maintenance was made during this period. This activity likely released plant wax* n*-alkanes into the air and influenced our samples.

In the time series plots of oxygenated aromatic compounds (Figures  S-3i, 3j, 3k, and 3l), their variations were different from those of other substances and extremely high concentrations of 1,2,4-BA and 4-Me-PhA were found in one sample only at Hamilton, July 3, and another at Simcoe, July 13. Although the explanation for July 13 at Simcoe would be the influence from the anthropogenic emissions found in other substances, we could not find any explanation for the high concentration of 1,2,4-BA at Hamilton.

## 4. Molecular Markers

### 4.1. PAHs

**Figure 2:**
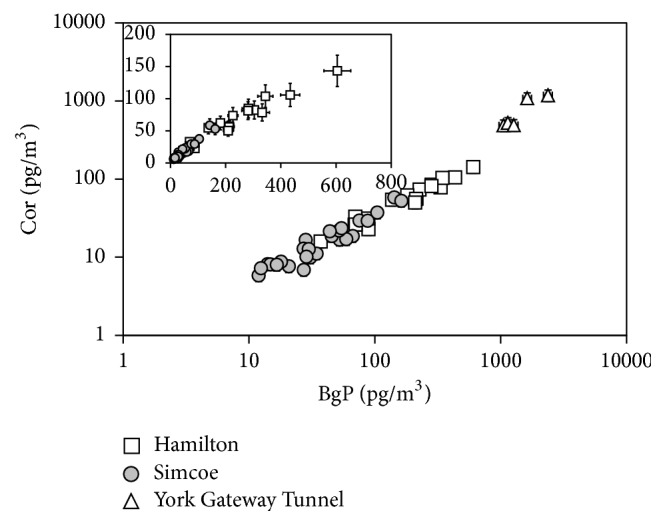
Scatter plot of coronene (Cor) concentration versus benzo[ghi]perylene (BgP) concentration.* Plots shown are in log-log scale and the inset shown is in expanded linear scale. The slopes of the linear regressions for Hamilton, Simcoe, and YGT are 0.27 ± 0.01 (r*^2^* = 0.914), 0.37 ± 0.01 (r*^2^* = 0.933), and 0.51 ± 0.04 (r*^2^* = 0.802), respectively.* Error bars shown are standard errors estimated from the replicate measurements.

Overall, BaP, Ind, BgP, and Cor concentrations at all three sites seemed to be highly correlated with each other (*r*^2^ ranging from 0.77 to 0.96), possibly implying contribution from a single source (e.g., Figure 2). However, the detailed analysis (*F*-test of one-sided analysis of variance) on slopes of the linear regressions for each site and concentration ratios of two species for each sample revealed that some PAHs showed statistically the same trend (or concentration ratios) at all three sites, but some did not. For example, concentration plots of BaP versus Ind [21] exhibited a high correlation, and the calculated *F*-value was 0.664. For the total number of 46 data points obtained from the three locations, the *α* threshold of 5% confidence interval for the null hypothesis is 3.2, which is apparently higher than the calculated *F*-value, justifying that the data from the three locations can be handled together. Thus, the slope ± standard error of the mean (SE) of the linear regression for BaP and Ind concentration plots including all the data from the three sites, 0.73 ± 0.02, represents the overall BaP/Ind ratio in the southern Ontario air. The single trend suggests that there was only one dominant and similar source for Ind and BaP at Hamilton and Simcoe, which is possibly the vehicular emissions, or that weights of contributions from the emission sources were, by coincidence, almost the same every day during the study period. Additionally, such a high correlation also implies that these PAHs were fresh because reactive PAHs including Ind and BaP deplete at different rates in the atmosphere [35–39], resulting in more scattered plot of these concentrations.

In contrast, the same analysis on Cor versus BgP for the three sites' data (Figure 2) resulted in *F*-value of 12.4, indicating that the trends of three sites are significantly different. Even the exclusion of YGT data, which show significantly different ratios in Figure 2, resulted in *F*-value of 7.6. The *α* threshold of 5% confidence interval for the null hypothesis of 1 × 43 degrees of freedom is 4.1. Thus, we concluded that the slopes of linear regressions for Cor and BgP concentrations at Hamilton (0.270 ± 0.008), Simcoe (0.37 ± 0.01), and YGT (0.51 ± 0.04) should be treated separately, meaning that the major emission sources for Cor and BgP are different at these sites.

To identify their sources, the BaP/Ind (0.73 ± 0.02 for all sites) and Cor/BgP (0.270 ± 0.008, 0.37 ± 0.01, and 0.51 ± 0.04 for Hamilton, Simcoe, and YGT, resp.) were compared with those ratios from the selected literatures reporting the four PAH concentrations from the source studies (Table 3). In this comparison, each concentration was normalized by the sum of the four PAH concentrations. The comparison showed that our BaP/Ind ratio statistically agrees with the ratio for diesel car emission [16]; thus, we conclude that diesel car emission was the major contributor of BaP and Ind in the southern Ontario air. The Cor/BgP ratio at YGT was similar to the ratios for the tunnel studies at Baltimore [14] and statistically agreed with gasoline car emission [16], wood burning [16], and emissions from an incinerator [15]. Because we expect that car emissions were predominant at YGT, the gasoline car emissions were likely the major contributor of Cor and BgP at YGT. The Cor/BgP ratios at Hamilton and Simcoe were similar to those of bituminous coal combustion [19] and diesel car emissions [16], respectively. An industry was located near the Hamilton site and there were tractor emissions in the agricultural field near the Simcoe site; therefore, these identifications are plausible. However, it should be noted that there was a coal-fired power plant (Nanticoke power plant) a few kilometers away from the Simcoe site. An impact from this power plant was observed in hopanes (discussed in the following section); thus, we could not rule out the possibility of its significant contribution in Cor and BgP emissions at Simcoe.

### 4.2. Hopanes

**Figure 3:**
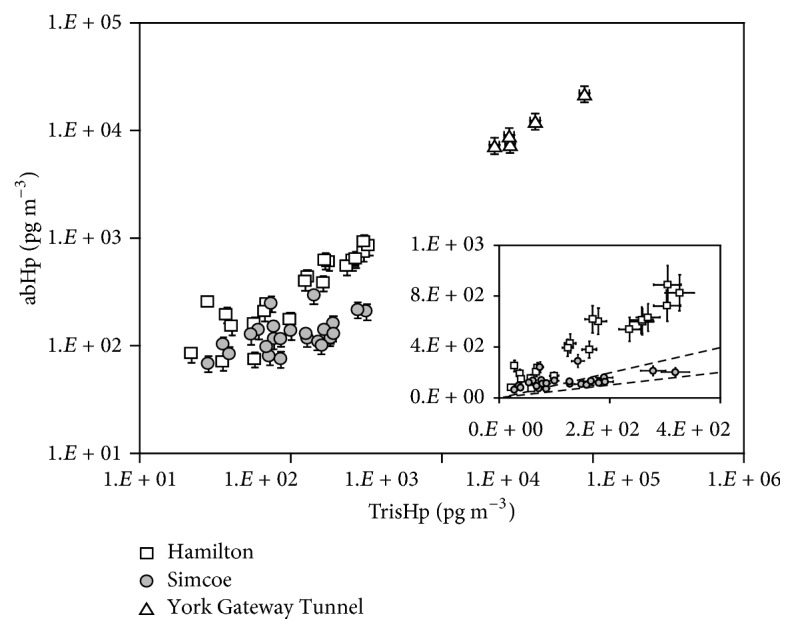
Scatter plot of *α*,*β*-hopane (abHp) concentration versus trisnorhopane (TrisHp) concentration in PM_10_ collected at three different locations.* The figure is in logarithm scale, and the inset is in expanded linear scale. The linear regression slopes for the Hamilton, Simcoe, and York Gateway Tunnel samples are 2.6 ± 0.1 (r*^2^* = 0.884), 0.89 ± 0.09 (r*^2^* = −0.579), and 2.7 ± 0.1 (r*^2^* = 0.953), respectively. Dotted lines shown in the inset are y = x and y = 0.5x, corresponding to the highest and the lowest ratios of α*,*β-hopane emission rate to trisnorhopane emission rate for coal combustions studied [19].* Error bars shown are standard errors estimated from the replicate measurements.

Plots of atmospheric concentrations of abHp and TrisHp (Figure 3) showed that the data points for Hamilton and YGT showed high correlations (*r*^2^ of 0.880 and 0.953, resp.). Their slopes agreed within the uncertainties (2.6 ± 0.1 for Hamilton and 2.7 ± 0.1 for YGT). It is believed that the origin of hopanes from car emissions is microdroplets of engine oil [4]; therefore, we conclude that car emissions were the major sources of abHp and TrisHp at Hamilton. It has been reported that abHp ratio to TrisHp in the emission from gasoline vehicles was in the range from 1.4 to 7.1, while diesel emissions showed the ratio of 3.5 [40]. This suggests that hopanes cannot distinguish between gasoline and diesel car emissions. The concentrations of abHp and TrisHp at Simcoe were not highly correlated but exhibited clear distinguishable trend as the concentrations were high. The slope of linear regression was 0.89 ± 0.09, significantly lower than the ratio of abHp to TrisHp from vehicular emissions discussed above. Oros and Simoneit [19] report that the ratios of abHp to TrisHp for lignite coal, brown coal, subbituminous coal, and bituminous coal were 0.5, 1.0, 0.8, and 0.7, respectively. When the impact of the plume from the Nanticoke provincial power plants was visually observed at Simcoe on July 11, the abHp/TrisHp ratio in the sample was 0.81. This agrees with the literature ratio of subbituminous and bituminous coals. Indeed, a report of the provincial government of Ontario states that the Nanticoke power plants used bituminous coals [41]. Although there were some periods where the abHp/TrisHp ratios from vehicular emissions were observed, the levels of hopane concentrations were small. Thus, we concluded that, at Simcoe, the Nanticoke power plants were likely responsible for the high concentration episodes of hopane series, and there was minor contribution from vehicular emissions.

### 4.3. *n*-Alkanes

**Figure 4:**
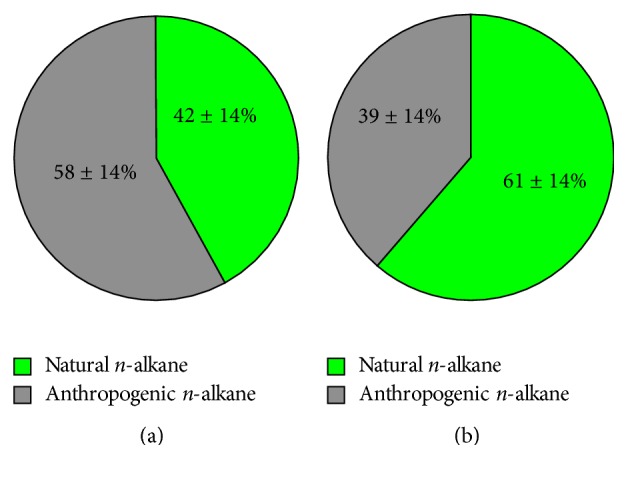
Mean percentage ± standard deviation of plant wax originated and petroleum originated particulate* n*-alkanes at Hamilton and Simcoe during SONTAS 2000.

When the series of* n*-alkanes satisfies CPI = 1, which is the indication of the origin from petroleum-related emissions, the concentration of plant wax* n*-alkane with *n* carbons ([C_*n*_]_plantwax_) can then be estimated by the following equation [42, 43]:

(2) $$\begin{array}{c} {\left[\;C_{n}\;\right]}_{plantwax}={\left[\;C_{n}\;\right]}_{obs}-\frac{\left\{{\left[C_{n-1}\right]}_{obs}+{\left[C_{n+1}\right]}_{obs}\right\}}{2}, \end{array}$$

where *n* is the carbon number of* n*-alkane and [C_*n*_]_obs_ is the concentration of* n*-alkane with the *n*th carbon. The equation assumes that the* n*-alkanes were originated only from petroleum-related emissions and plant wax and assumes that all even number* n*-alkanes were originated from petroleum emission. Results showed that the contributions of plant wax* n*-alkanes at Hamilton and Simcoe (average ± SD) were 42 ± 14% and 61 ± 14%, respectively (Figure 4). With respect to the concentrations, on average, the sum of particulate* n*-alkane concentrations (±SD) at Hamilton and Simcoe was 11300 ± 3900 pg m^−3^ and 8700 ± 2000 pg m^−3^, respectively. The concentration at Simcoe was slightly lower than the concentration at Hamilton, and this can be explained as more contribution from man-made emissions at Hamilton. Nevertheless, the magnitudes of* n*-alkane concentrations at both sites are similar; therefore, the observed levels are more likely the background concentration of airborne particulate plant wax* n*-alkane (C_20_–C_34_) in the southern Ontario air.

### 4.4. Oxygenated Aromatic Compounds

Oxygenated aromatic compounds in airborne POM can be primary [44, 45] and secondary products [5, 46, 47]. The high concentrations of these compounds observed at YGT suggest the possibility of their primary origins from vehicular emissions. Scatter plots between the oxygenated aromatic compounds at Hamilton and Simcoe all together gave more detailed ideas on their emission sources.

**Figure 5:**
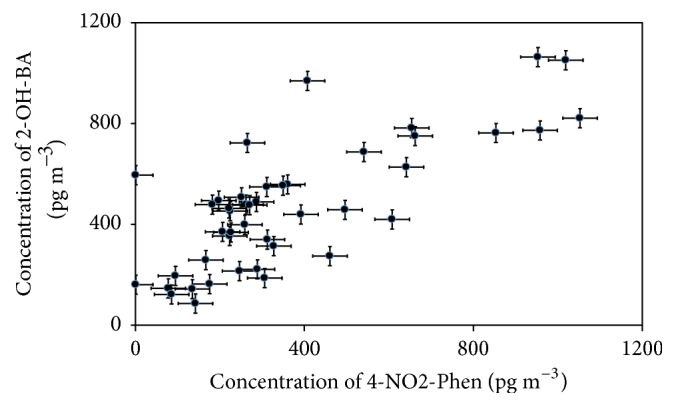
Scatter plot of 4-nitrophenol (4-NO2-Phen) concentration versus 2-hydroxybenzoic acid (2-OH-BA) concentration. Error bars shown are the 3% reproducibility of CE measurements.

First, 4-NO_2_-Phen showed a proportional increase with 2-OH-BA (the highest correlation with* r*^2^ = 0.855), indicating that 4-NO_2_-Phen and 2-OH-BA were from similar source(s) at Hamilton and Simcoe (Figure 5). Second, excluding the July 13 data due to the apparent influence from the coal-fired power plant at Simcoe, the concentration ratios of Hamilton to Simcoe were significantly larger in 3-OH-BA (12 ± 6, average ± SE), 4-OH-BA (13 ± 3), and 4-Me-PhA (13 ± 3) than those in the other oxygenated aromatic compounds (1.0 ± 0.3~2.6 ± 0.9), suggesting local emission sources for 3-OH-BA, 4-OH-BA, and 4-Me-PhA at Hamilton. Third, on July 13, the 3-OH-BA, 4-OH-BA, and 4-Me-PhA concentration levels at Simcoe reached high level similar to the level observed at Hamilton, indicating that coal-fired power plant was their emission source at Simcoe. Fourth, overall, the* o*-PhA,* m*-PhA, and* p*-PhA and 1,2,3-BA, 1,2,4-BA, and 1,2,4,5-BA concentrations at Hamilton and Simcoe were almost in the same levels and were highly correlated (*r*^2^ = 0.627 or higher). This may imply that the southern Ontario air contained those compounds as background, possibly secondary products.

### 4.5. Wind Sector Dependence

Hourly averaged wind sectors were compared with the hourly averaged SO_2_ mixing ratios or daily averaged molecular marker concentrations. In this evaluation, we used wind sectors (from 1° to 360° with the north at 360°) whose wind speeds were equivalent to or larger than 4 km h^−1^. This filtration resulted in the following: in total, 63% and 42% of observed hourly wind sectors were used for the Hamilton and Simcoe data, respectively.

**Figure 6:**
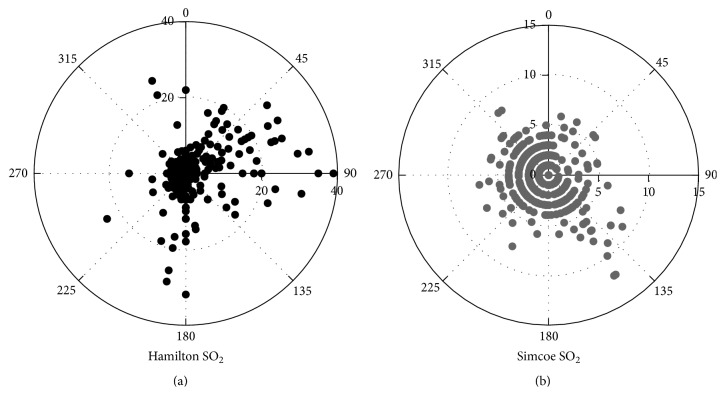
Polar plot of hourly averaged SO_2_ mixing ratios at (a) Hamilton and (b) Simcoe. Radial axis shown is in ppbv.

There was clear wind sector dependence on SO_2_ mixing ratios at Hamilton (Figure 6(a)) and Simcoe (Figure 6(b)). High SO_2_ mixing ratios were observed as wind was from the directions of 30°–90° and 170°–190° at Hamilton. The influence from the direction of 30°–90° was observed over the six days during the campaign. It has been reported for the same field study at the same site that the size distribution measurements of PM also showed high concentrations of PM in Aitken mode as wind was from the direction of 30°–90° [48]. In this literature, it was concluded that this sector dependence was due to the emission from the industry located ~3 km northeast to east of the site. Another clear wind sector dependency, 170°–190°, was observed only for a short time period (9:00 to 15:00 on June 24). This episode lasted only for 7 hours. Possible sources in the southward are diesel fuelled mobile sources from downtown Hamilton. A clear wind sector dependency was observed at Simcoe as wind was from 120°–150°, but the magnitude was low (up to 12 ppb). As demonstrated by other substances, this was influence from the Nanticoke power plants.

For comparison of the hourly averaged wind sectors with the daily averaged molecular marker concentrations, we used conditional probability function (CPF) [49]:

(3) $$\begin{array}{c} CPF=f_{\Delta \theta }\times \frac{m_{\Delta \theta }}{n_{\Delta \theta }}. \end{array}$$

In this equation, *n*_Δ*θ*_ is the total frequency of occurrence of the wind sector between *θ* and *θ* + Δ*θ* during the whole field study period, *m*_Δ*θ*_ is the frequency of occurrence of the wind sector during the collection of samples exceeding a threshold concentration, and *f*_Δ*θ*_ is a weighing factor [50]. Instead of analyzing CPF for individual substance, we performed the analysis for the sum of the same chemical species (*i.e.*, the sum of concentrations of PAHs, hopanes,* n*-alkanes, or the groups of oxygenated aromatic compounds referred to in the previous subsection). We also used the upper 50 percentile concentration for the threshold concentration of each chemical homologue species to extract meaningful dependency and *f*_Δ*θ*_ of one and *f*_Δ*θ*_ of a square root of *n*_Δ*θ*_ over a square root of 10 were used if *n*_Δ*θ*_ > 10 and *n*_Δ*θ*_ ≤ 10 [48], respectively. The resolution of wind sector, Δ*θ*, was set to 20° in our CPF analysis.

**Figure 7:**
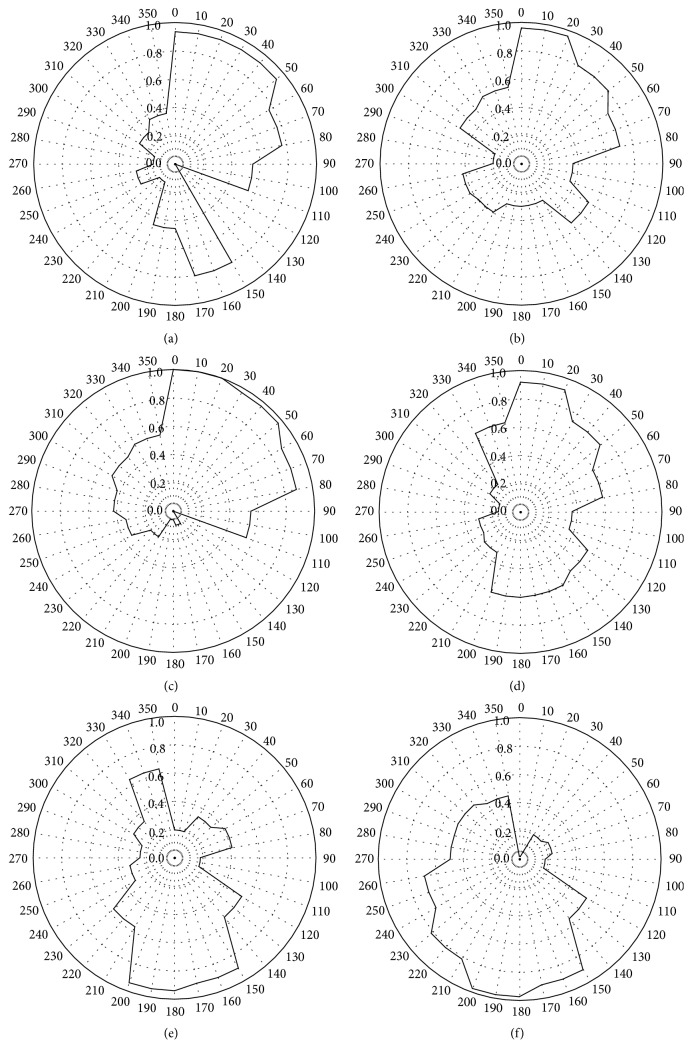
Rose plot of CPF for (a) the sum of PAHs, (b) the sum of hopanes, (c) the sum of* n*-alkanes, (d) the sum of 4-NO2-Phen and 2-OH-BA, (e) the sum of 3-OH-BA, 4-OH-BA, and 4-Me-PhA, and (f) the sum of* o*-PhA,* m*-PhA,* p*-PhA, 1,2,3-BA, 1,2,4-BA, and 1,2,4,5-BA at Hamilton.

The plots of CPF for the PAH, hopane, and* n*-alkane concentrations during SONTAS 2000 at Hamilton (Figures 7(a)–7(f)) showed high concentrations as wind was from the direction of 360° to 90° (northerly to easterly), indicating the impact from the emissions from the industry and/or from traffic emissions from the highway of Queen Elizabeth Way (the north to northeast of the sampling station). There was another clear influence from the southeasterly wind on the PAH concentrations, possibly indicating traffic emissions in downtown Hamilton. The CPF plots for the oxygenated aromatic compounds showed that the sum of 4-NO_2_-Phen and 2-OH-BA and the sum of 3-OH-BA, 4-OH-BA, and 4-Me-PhA had also the similar wind sector dependence to the primary emission markers. However, the sum of* o*-PhA,* m*-PhA,* p*-PhA, 1,2,3-BA, 1,2,4-BA, and 1,2,4,5-BA was dependent on southerly wind, indicating that their origins are different.

**Figure 8:**
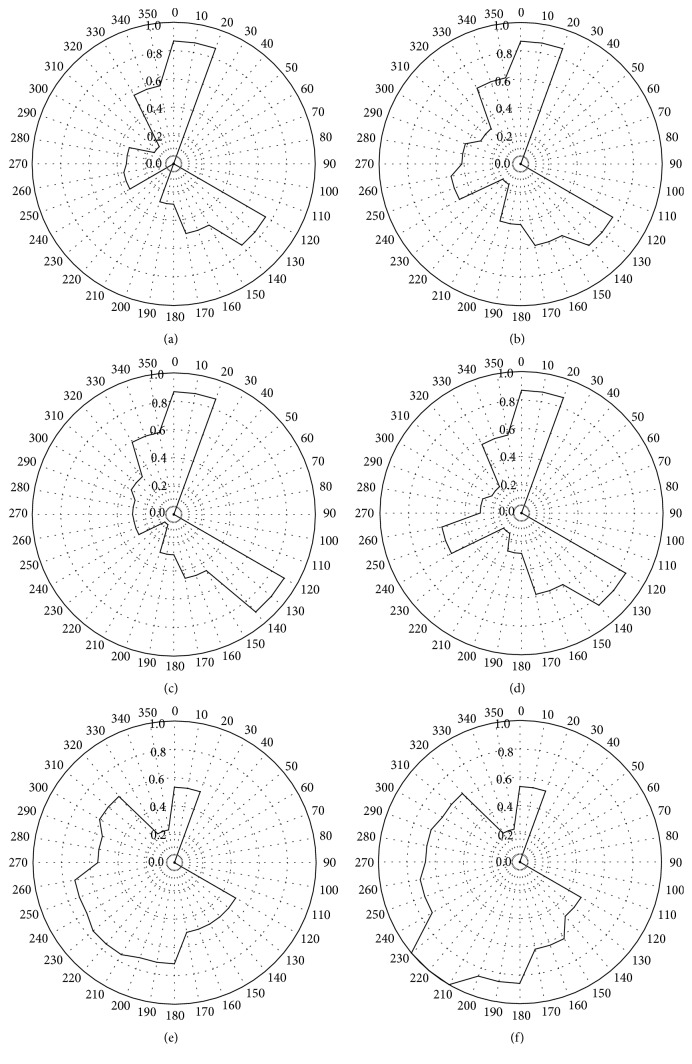
Rose plot of CPF for (a) the sum of PAHs, (b) the sum of hopanes, (c) the sum of* n*-alkanes, (d) the sum of 4-NO2-Phen and 2-OH-BA, (e) the sum of 3-OH-BA, 4-OH-BA, and 4-Me-PhA, and (f) the sum of* o*-PhA,* m*-PhA,* p*-PhA, 1,2,3-BA, 1,2,4-BA, and 1,2,4,5-BA at Simcoe.

The CPF analysis for the Simcoe data (Figures 8(a)–8(f)) showed that the concentrations of PAHs, hopanes,* n*-alkanes, 4-NO_2_-Phen, and 2-OH-BA were high as the wind sectors were from 330°–20° (*i.e.*, northerly wind) and 120°–170° (*i.e.*, southeasterly wind). We could not identify this emission source located in the north of the sampling site at Simcoe. Chan and Mozurkewich [48] also found in this study that fine PM was likely transported from Toronto to the Simcoe sampling site; therefore, this long-range transport may be the explanation. The high concentrations from the southeasterly direction are explained by the influence from the coal-fired power plant. The CPF plots for the sum of 3-OH-BA, 4-OH-BA, and 4-Me-PhA and the sum of* o*-PhA,* m*-PhA,* p*-PhA, 1,2,3-BA, 1,2,4-BA, and 1,2,4,5-BA (polycarboxylic acids) showed different dependency, but both did not exhibit the similar trend to the primary emission markers, such as PAHs and hopanes. Their sources are likely different from the primary emissions. It is interesting to see that the group of 3-OH-BA, 4-OH-BA, and 4-Me-PhA and the group of polycarboxylic acids showed similar dependency for the polycarboxylic acids observed at Hamilton. This is consistent with our previous conclusion that* o*-PhA,* m*-PhA,* p*-PhA, 1,2,3-BA, 1,2,4-BA, and 1,2,4,5-BA observed were contained in the air mass covering southern Ontario.

### 4.6. APCA

**Figure 9:**
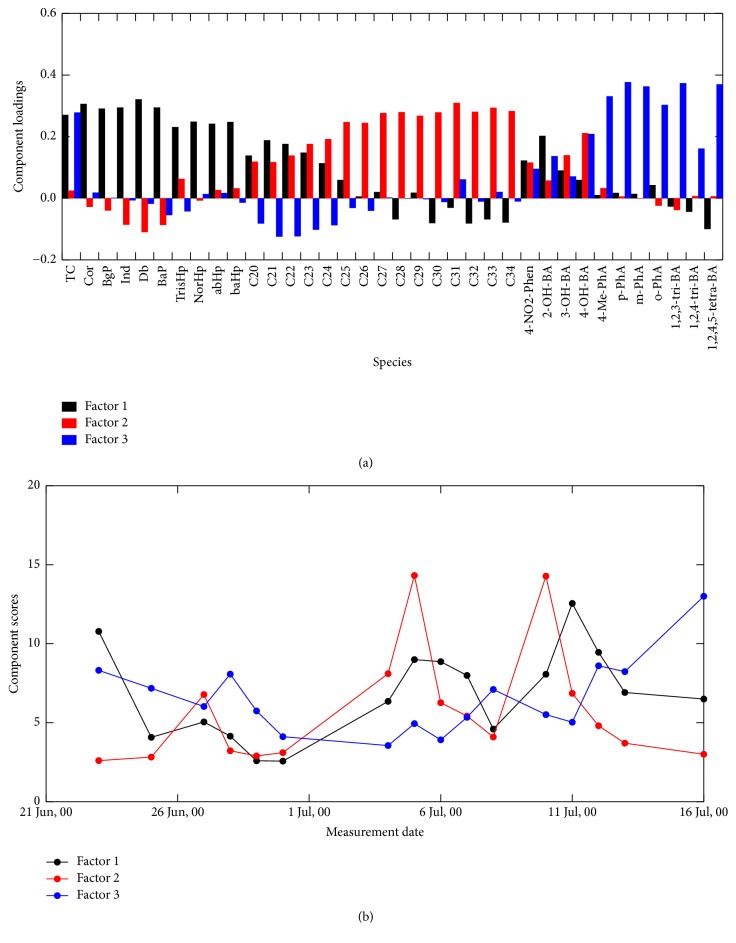
(a) Loading of three factorial analyses and (b) time series score of three loadings for the Hamilton dataset.

**Figure 10:**
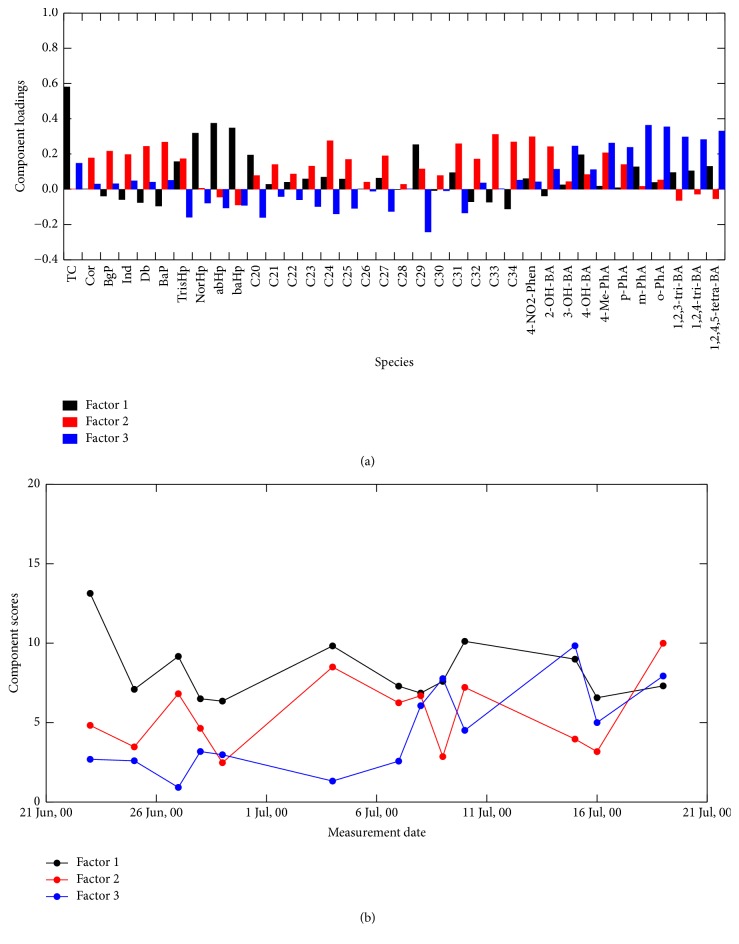
(a) Loading of three factorial analyses and (b) time series score of three loadings for the Simcoe dataset.

Scree plots of our APCA for up to ten loadings (or components) indicated more stable eigenvalues at Hamilton and Simcoe as more than three components were retained (Figures  S-4a and 4b). It was found that the analysis with more than 3 loadings seemed to unnecessarily deconvolute the molecular marker signatures. To determine plausible number of loadings, we considered the following findings previously discussed. (1) From the analysis of PAH and hopane markers, vehicular emissions and coal (or similar petroleum fuel) combustion were more likely the major emissions sources at both sites. (2) At both sites, 4-NO_2_-Phen and 2-OH-BA were originated from different emission source(s) compared to the other oxygenated aromatic compounds. (3) The emission source for 3-OH-BA, 4-OH-BA, and 4-Me-PhA at Hamilton was different from their source at Simcoe, which is more likely the Nanticoke power plant. (4)* o*-PhA,* m*-PhA,* p*-PhA, 1,2,3-BA, 1,2,4-BA, and 1,2,4,5-BA were in the same concentration level at both sites and those can be of the same origin, secondary POM in the southern Ontario air. With consideration of these findings, we concluded that three factorial solutions of APCA seem to be the most plausible at Hamilton (Figure 9) and Simcoe (Figure 10). According to the marker distribution shown in Figure 9(a), plots of grey, red, and blue are vehicular emissions, unknown, and secondary products at Hamilton, respectively. Meanwhile, plots of grey, red, and blue at Simcoe seem to be coal combustion, vehicular emissions, and secondary products, respectively (Figure 10(a)). The results also demonstrated the limitation of APCA that the analysis could not differentiate vehicular emission and coal combustion at both sites; therefore, it should be noted that the plot in grey at both sites likely includes both sources. There was another problem that the plot in red at Hamilton, which is accompanied with* n*-alkanes and some of the oxygenated aromatic compounds, could not be identified. Since the majority of* n*-alkanes at Hamilton were expected to be the origin of vehicular emissions, the APCA seems to unsuccessfully deconvlute the emission sources for PAHs, hopanes, and* n*-alkanes at Hamilton. Those problems may be attributed to the fact that the number of our data points was limited. APCA on large datasets with respect to observations for longer time period would probably improve the results. Nevertheless, the combination of the plots in grey and red at both sites likely represents anthropogenic emissions, while the plot in blue represents the secondary POM. Time series plots of their scores (Figure 9(b) for Hamilton and Figure 10(b) for Simcoe) show that primary man-made emissions are the major source of POM in the southern Ontario air during the study period. However, it is worthwhile to note that, similarly to the late of the study period at both sites, the burden of polycarboxylic acids sometime became large. Combination of oxygenated aromatic compounds with VOC studies may gain a deeper insight into the production of secondary POM in the southern Ontario air.

## 5. Summary

Molecular marker analysis here demonstrated that PAHs, hopanes, and* n*-alkanes give valuable information of primary sources, particularly for vehicular emissions and coal combustion, while oxygenated aromatic compounds can be primary and/or secondary products. The paper here also demonstrated that, with combination of wind sector analysis and principal component analysis, the molecular marker can give more convincing evidence for source identification.

## Supplementary Material

Figure S-1: Daily frequency of wet deposition occurrence (rain and fog) during the SONTAS 2000 in Hamilton. The frequency was determined by dividing the number of hourly counts of wet deposition during the sampling by the sampling duration (h).
Figure S-2: Hourly averaged SO2 mixing ratio at (a) Hamilton and (b) Simcoe.
Figure S-3: Time series plot of TC and molecular markers in PM10 collected at Hamilton (left) and the Simcoe (right). Error bars shown are standard errors estimated based on the replicate measurements.
Figure S-4: Plot of square root of the sum of unused eigenvalues as a function of number of loading (scree plot) for (a) Hamilton and (b) Simcoe.

## Figures and Tables

**Table 1:** Mean of molecular marker concentration and concentration range observed during the SONTAS 2000.

Location	Hamilton (*n* = 28)	Simcoe (*n* = 34)	Toronto Tunnel (*n* = 5)
Ext. method	Soxhlet and sonication	Soxhlet and sonication	Soxhlet and sonication
Sample type	PM_10_	PM_10_	PM_10_
Filter media ^a^	Quartz filter	Quartz filter	Quartz filter
Sampling time	24 h	24 h	4h
		(ng m^−3^)	
*PAHs*			
Benzo[a]pyrene (BaP)	0.105 (0.009–0.608)	0.023 (<DL–0.133)	0.513 (0.165–1.212)
Dibenz[a,h]anthracene (Db)	0.030 (0.004–0.131)	0.016 (0.001–0.073)	0.075 (0.005–0.162)
Indeno[1,2,3-cd]pyrene (Ind)	0.184 (0.019–0.804)	0.070 (0.001–0.447)	0.659 (0.230–1.501)
Benzo[ghi]perylene (BghiP)	0.175 (0.023–0.605)	0.049 (0.011–0.265)	1.495 (1.054–2.380)
Coronene (Cor)	0.052 (0.014–0.143)	0.019 (0.006–0.082)	0.754 (0.488–1.181)
*Hopanoids*			
17*α*(H)-22,29,30-Trisnorhopane (TrisHp)	0.132 (0.022–0.326)	0.181 (0.028–0.530)	4.166 (2.234–8.811)
17*β*(H),21*α*(H)-30-Norhopane (NorHp)	0.044 (0.004–0.111)	0.019 (0.007–0.052)	1.000 (0.640–1.747)
17*α*(H),21*β*(H)-Hopane (abHp)	0.342 (0.043–0.887)	0.154 (0.068–0.445)	11.788 (7.394–22.476)
17*β*(H),21*α*(H)-Hopane (baHp)	0.072 (0.007–0.190)	0.014 (0.006–0.042)	1.289 (0.838–2.309)
*n-Alkanes*			
Eicosane (C20)	1.150 (<DL–4.370)	1.216 (<DL–6.036)	59.297 (45.454–97.709)
Heneicosane (C21)	1.835 (0.241–6.425)	1.351 (0.058–6.855)	40.047 (22.276–54.842)
Docosane (C22)	1.410 (0.222–3.625)	1.288 (0.066–6.020)	35.134 (14.490–56.449)
Tricosane (C23)	2.738 (0.384–6.309)	2.414 (0.272–10.399)	31.766 (11.235–69.772)
Tetracosane (C24)	1.363 (0.231–4.634)	0.704 (0.119–2.249)	18.945 (7.760–41.957)
Heptacosane (C25)	2.159 (0.473–6.264)	1.608 (0.274–10.980)	17.462 (5.061–40.785)
Hexacosane (C26)	1.116 (0.007–4.967)	0.642 (0.081–12.471)	9.431 (4.523–17.813)
Heptacosane (C27)	3.092 (1.007–9.058)	2.210 (0.467–15.265)	16.125 (8.581–29.425)
Octacosane (C28)	1.469 (0.065–6.889)	0.968 (<DL–21.051)	11.115 (5.559–15.220)
Nonacosane (C29)	3.790 (1.149–9.589)	3.175 (0.351–10.378)	14.042 (8.656–22.051)
Triacontane (C30)	1.383 (0.130–7.528)	0.504 (<DL–8.762)	10.923 (7.053 -15.800)
Hentriacontane (C31)	4.160 (1.913–10.451)	2.700 (0.940–11.769)	27.290 (13.958–46.388)
Dotriacontane (C32)	0.611 (<DL–3.205)	0.254 (<DL–3.798)	10.498 (5.550–29.022)
Tritriacontane (C33)	0.992 (0.345–3.801)	0.518 (0.038–3.617)	12.297(5.663–22.460)
Tetratriacontane (C34)	0.286 (<DL–1.352)	0.190 (<DL–1.689)	6.774 (3.591–9.015)
*Oxygenated aromatic compounds*			
4-Nitrophenol (4-NO2-Phen)	0.727 (0.204–1.667)	0.408 (<DL–1.840)	9.471 (2.599–25.090)
2-Hydroxybenzoic acid (2-OH-BA)	0.724 (0.204–1.473)	0.545 (0.086–1.827)	13.774 (4.367–38.145)
3-Hydroxybenzoic acid (3-OH-BA)	0.144 (<DL–0.467)	0.052 (<DL–0.469)	6.298 (2.144–18.535)
4-Hydroxybenzoic acid (4-OH-BA)	0.687 (0.349–1.096)	0.272 (0.022–0.988)	9.963 (2.201–32.593)
4-Methylphthalic acid (4-Me-PhA)	2.719 (1.287–6.420)	1.245 (0.103–6.706)	17.871 (5.490–44.862)
Para-phthalic acid (*p*-PhA)	1.666 (0.660–3.385)	1.452 (0.288–3.716)	11.667 (2.980–34.641)
Meta-phthalic acid (*m*-PhA)	0.487 (0.209–1.158)	0.358 (0.145–0.775)	9.232 (1.410–29.403)
Ortho-phthalic acid (*o*-PhA)	10.481 (<DL–23.689)	11.672 (4.664–22.977)	53.311 (18.578–162.944)
1,2,3-Tricarboxylic benzoic acid (1,2,3-BA)	2.725 (0.731–4.837)	3.029 (0.814–8.409)	15.760 (1.434–52.148)
1,2,4-Tricarboxylic benzoic acid (1,2,4-BA)	3.307 (1.194–16.006)	2.460 (0.796–5.831)	23.465 (2.540–67.539)
1,2,4,5-Tetracarboxylic benzoic acid (1,2,4,5-BA)	0.579 (0.220–1.508)	0.391 (0.059–0.909)	5.808 (0.523–17.536)

The mean values of atmospheric concentrations observed are given with either the ranges (two values in a bracket) or the standard deviations (a single value in a bracket).

**Table 2:** Molecular marker concentrations observed in other locations.

Location	Hamilton 1990^a^ (*n* = 33)	Philadelphia 1999^b^ (*n* = 41)	Beijing^c^
Ext. method	Soxhlet	Soxhlet	Sonication
Sample type	PM_10_	PM_10_	PM_10_
Filter media^a^	T	Q	G or Q
Sampling time	24 h	10 h	24 h
		(ng m^−3^)^b^	

*PAHs*			
Benzo[a]pyrene (BaP)	2.0 (0.01–12.9)	—	45.6
Indeno[1,2,3-cd]pyrene (Ind)	3.0 (0.03–21.2)	—	30.1
Benzo[ghi]perylene (BghiP)	4.3 (0.07–25.4)	—	33.8
Coronene (Cor)	—	—	—
*Hopanoids*			
17*α*(H)-22,29,30-Trisnorhopane (TrisHp)	—	0.107 (0.068)	12.1
17*β*(H),21*α*(H)-30-Norhopane (NorHp)	—	—	16.6
17*α*(H),21*β*(H)-Hopane (abHp)	—	0.317 (0.151)	26.4
17*β*(H),21*α*(H)-Hopane (baHp)	—	—	13.2
*n-Alkanes*			
Eicosane (C20)	—	—	102.1
Heneicosane (C21)	—	—	146.2
Docosane (C22)	—	—	170.4
Tricosane (C23)	—	0.32 (0.21)	180.9
Tetracosane (C24)	—	0.55 (0.57)	148.4
Heptacosane (C25)	—	1.60 (1.29)	128.1
Hexacosane (C26)	—	0.85 (1.09)	80.6
Heptacosane (C27)	—	1.90 (1.16)	60.1
Octacosane (C28)	—	0.670 (1.02)	32.4
Nonacosane (C29)	—	2.94 (1.89)	42.8
Triacontane (C30)	—	0.49 (0.74)	20.2
Hentriacontane (C31)	—	2.32 (1.75)	24.9
Dotriacontane (C32)	—	—	8.8
Tritriacontane (C33)	—	—	9.3
Tetratriacontane (C34)	—	—	nd
*Oxygenated aromatic compounds*			
4-Nitrophenol (4-NO2-Phen)	—	—	—
2-Hydroxybenzoic acid (2-OH-BA)	—	—	—
3-Hydroxybenzoic acid (3-OH-BA)	—	—	—
4-Hydroxybenzoic acid (4-OH-BA)	—	—	—
4-Methylphthalic acid (4-Me-PhA)	—	—	—
Para-phthalic acid (*p*-PhA)	—	—	—
Meta-phthalic acid (*m*-PhA)	—	—	—
Ortho-phthalic acid (*o*-PhA)	—	—	—
1,2,3-Tricarboxylic benzoic acid (1,2,3-BA)	—	—	—
1,2,4-Tricarboxylic benzoic acid (1,2,4-BA)	—	—	—
1,2,4,5-Tetracarboxylic benzoic acid (1,2,4,5-BA)	—	—	—

Location	Houston 1997^d^ (*n* = 13)	Atlanta 1999^e^	L.A. Tunnel^f^
Ext. method	Sonication	Sonication	Sonication
Sample type	PM_2.5_	PM_2.5_	
Filter media	Q	Q	Q
Sampling time	24 h	24 h	4 h
		(ng m^−3^)^a^	(*μ*g L^−1^)

*PAHs*			
Benzo[a]pyrene (BaP)	0.02	0.18	18.3
Indeno[1,2,3-cd]pyrene (Ind)	<DL	0.26	30.6
Benzo[ghi]perylene (BghiP)	0.01	0.55	102.2
Coronene (Cor)	—	0.26	—
*Hopanoids*			
17*α*(H)-22,29,30-Trisnorhopane (TrisHp)	0.19	0.10	18.1
17*β*(H),21*α*(H)-30-Norhopane (NorHp)	—	—	—
17*α*(H),21*β*(H)-Hopane (abHp)	0.07	0.56	82.0
17*β*(H),21*α*(H)-Hopane (baHp)	—	—	—
*n-Alkanes*			
Eicosane (C20)	0.29	—	48.9
Heneicosane (C21)	0.43	—	113.8
Docosane (C22)	0.61	—	123.9
Tricosane (C23)	0.68	—	164.5
Tetracosane (C24)	2.45	3.97	229.2
Pentacosane (C25)	1.58	4.00	158.4
Hexacosane (C26)	2.66	2.87	146.7
Heptacosane (C27)	1.95	2.55	104.0
Octacosane (C28)	1.96	1.41	77.6
Nonacosane (C29)	3.04	2.36	128.3
Triacontane (C30)	1.45	0.87	82.2
Hentriacontane (C31)	3.11	1.88	93.4
Dotriacontane (C32)	1.28	0.42	48.4
Tritriacontane (C33)	2.37	0.50	33.7
Tetratriacontane (C34)	—	—	
*Oxygenated aromatic compounds*			
4-Nitrophenol (4-NO2-Phen)	—	—	—
2-Hydroxybenzoic acid (2-OH-BA)	—	—	—
3-Hydroxybenzoic acid (3-OH-BA)	—	—	—
4-Hydroxybenzoic acid (4-OH-BA)	—	—	—
4-Methylphthalic acid (4-Me-PhA)	—	1.94	—
Para-phthalic acid (*p*-PhA)	—	5.98	—
Meta-phthalic acid (*m*-PhA)	—	0.64	—
Ortho-phthalic acid (*o*-PhA)	—	5.89	—
1,2,3-Tricarboxylic benzoic acid (1,2,3-BA)	—	—	—
1,2,4-Tricarboxylic benzoic acid (1,2,4-BA)	—	—	—
1,2,4,5-Tetracarboxylic benzoic acid (1,2,4,5-BA)	—	—	—

^a^Cited in Legzdins et al. [9].  ^b^Cited in Shihabut et al. [10].  ^c^Cited in Simoneit et al. (2007).  ^d^Cited in Fraser et al. [11] as the summer data from the HRM-3 site in Houston.  ^e^Cited in Zheng et al. [12] as the data from the Jefferson street in Atlanta.  ^f^Cited in Fraser et al. [13].

**Table 3:** Comparison for normalized concentration of PAHs with literature values.

Species	Our studies			Benner et al. [14]	Freeman and Cattell [15]			
	Soxhlet			Soxhlet	Sonication			
	Toronto Tunnel	Hamilton	Simcoe	Baltimore Tunnel	Bush fire	Leaf combustion	Incinerator^‡^	
	(*n* = 5)	(*n* = 20)	(*n* = 24)	(*n* < 27)				
BaP	0.14 ± 0.06	0.18 ± 0.06	0.14 ± 0.05	0.24 ± 0.06	0.69	0.57	0.23	
Ind	0.18 ± 0.06	0.35 ± 0.03	0.41 ± 0.04	0.20 ± 0.07	0.20	0.28	0.43	
BgP	0.46 ± 0.08	0.36 ± 0.03	0.31 ± 0.02	0.36 ± 0.13	0.10	0.14	0.21	
Cor	0.22 ± 0.06	0.11 ± 0.03	0.13 ± 0.03	0.20 ± 0.09	0.01	0.01	0.13	

Species	Li and Kamens [16]			Alsberg et al. [17]		Rogge et al. [18]		
	Soxhlet			Soxhlet		Sonication		
	Wood	Gasoline	Diesel	Gasoline B	Gasoline C	Gasoline 1^†^	Gasoline 2^†^	Diesel
	(*n* = 14)	(*n* = 5)	(*n* = 3)					

BaP	0.37 ± 0.08	0.18 ± 0.06	0.20 ± 0.06	0.12	0.14	0.15	0.23	0.45
Ind	0.28 ± 0.06	0.12 ± 0.03	0.31 ± 0.14	0.11	0.11	0.02	0.06	n.d.
BgP	0.23 ± 0.06	0.42 ± 0.08	0.36 ± 0.13	0.37	0.39	0.48	0.58	0.55
Cor	0.12 ± 0.03	0.29 ± 0.04	0.14 ± 0.05	0.41	0.36	0.35	0.13	n.d.

Species	Oros and Simoneit [19]		Simoneit et al. (2005)			Wornat et al. [20]		
	Sonication		Sonication			Sonication		
						(*n* = 8)		
	Coal combustion							
	Subbituminous	Bituminous	Plastic bags^§^	Roadside litter^§^	Landfill trash^§^	Residential coal combustion		

BaP	0.40	0.27	0.11	0.20	0.18	0.214 ± 0.062		
Ind	0	0.22	0.61	0.49	0.51	0.240 ± 0.093		
BgP	0.60	0.41	0.23	0.25	0.26	0.384 ± 0.077		
Cor	0	0.10	0.24	0.07	0.05	0.162 ± 0.025		

The values are given with standard deviations, if reported, except the data from Li and Kamens (±standard errors).  ^†^Gasoline 1 and Gasoline 2 are noncatalyst gasoline vehicles and catalyst vehicles, respectively.  ^§^The PM was from combustion of Chilean materials.  ^‡^Emissions were from the combustion of paper and cardboard.

## Nomenclature

PM: Particulate matter
PM_10_: Particulate matter smaller than 10 *μ*m in aerodynamic diameter
POM: Particulate organic matter
TC: Total carbon
CPI: Carbon preference index
APCA: Absolute principal component analysis
PAHs: Polycyclic aromatic hydrocarbons
BaP: Benzo[a]pyrene
Ind: Indeno[1,2,3-cd]pyrene
BgP: Benzo[ghi]perylene
Cor: Coronene
TrisHp: Trisnorhopane
NorHp: Norneohopane
abHp: *α*,*β*-Hopane
baHp: *β*,*α*-Hopane
C_20_ to C_34_: *n*-Alkanes with the carbon numbers from C_20_ to C_34_
4-NO_2_-Phen: 4-Nitrophenol
2-OH-BA: 2-Hydroxybenzoic acid
3-OH-BA: 3-Hydroxybenzoic acid
4-OH-BA: 4-Hydroxybenzoic acid
*o*-PhA: Ortho-phthalic acid
*m*-PhA: Meta-phthalic acid
*p*-PhA: Para-phthalic acid
4-Me-PhA: 4-Methylphthalic acid
1,2,3-BA: 1,2,3-Benzenetricarboxylic acid
1,2,4-BA: 1,2,4-Benzenetricarboxylic acid
1,2,4,5-BA: 1,2,4,5-Benzenetetracarboxylic acid.
