# Supplementary material for: Molecular Marker Study of Particulate Organic Matter in Southern Ontario Air
Source: J Anal Methods Chem. 2017 Sep 17;2017:3504274. doi: 10.1155/2017/3504274 (PMC5623806; doi:10.1155/2017/3504274)
Supplement: Supplementary file 1 — Figure S-1: Daily frequency of wet deposition occurrence (rain and fog) during the SONTAS 2000 in Hamilton. The frequency was determined by dividing the number of hourly counts of wet deposition during the sampling by the sampling duration (h). Figure S-2: Hourly averaged SO2 mixing ratio at (a) Hamilton and (b) Simcoe. Figure S-3: Time series plot of TC and molecular markers in PM10 collected at Hamilton (left) and the Simcoe (right). Error bars shown are standard errors estimated based on the replicate measurements. Figure S-4: Plot of square root of the sum of unused eigenvalues as a function of number of loading (scree plot) for (a) Hamilton and (b) Simcoe. [file 3504274.f1.docx]

Supporting Information for the Manuscript Entitled

“Molecular Marker Study of Particulate Organic Matter in Southern Ontario Air”

by Irei et al.

4 Pages

4 Figures

0 Tables


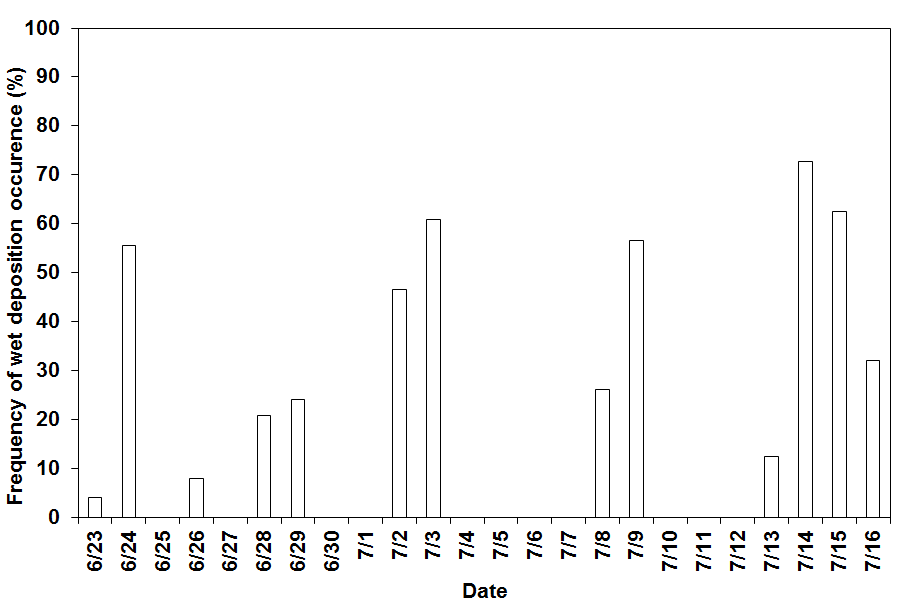


**Figure S-1.** Dairy frequency of wet deposition occurrence (rain and fog) during the SONTAS 2000 in Hamilton. The daily frequency was determined by the division of the number of hourly reports of precipitation by the sampling duration in hour.


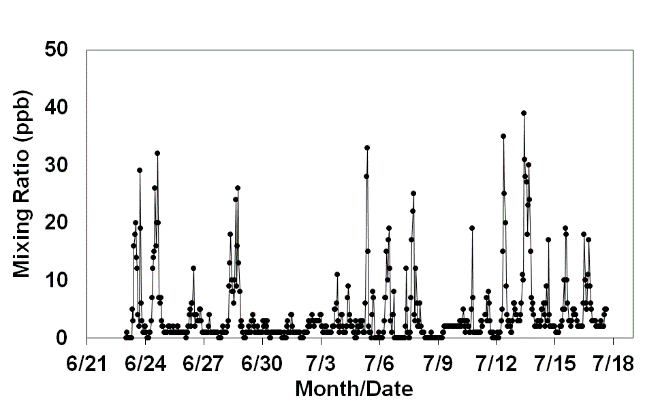

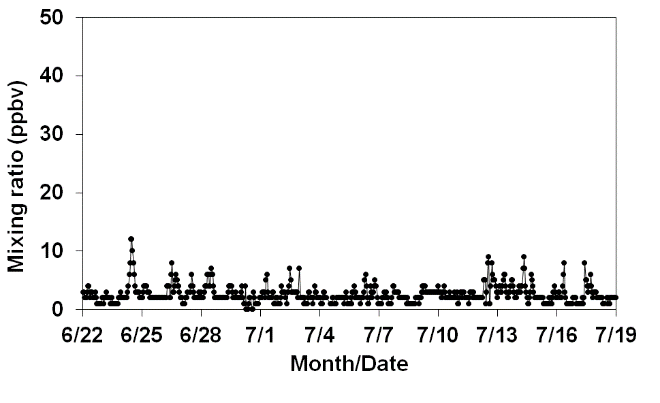


**(*a*)**

**(*b*)**

**Figure S-2.** Hourly averaged SO_2_ mixing ratio at (*a*) Hamilton and (*b*) Simcoe.

**(*b*)**

**(*a*)**

**(*d*)**

**(*c*)**

**(*f*)**

**(*e*)**

**(*h*)**

**(*g*)**

**(*j*)**

**(*i*)**

**(*l*)**

**(*k*)**

**Figure S-3.** Time series plot of TC and molecular markers in PM_10_ collected at Hamilton (*left*) and the Simcoe (*right*). Error bars shown are the errors of the means estimated based on the replicate measurements.


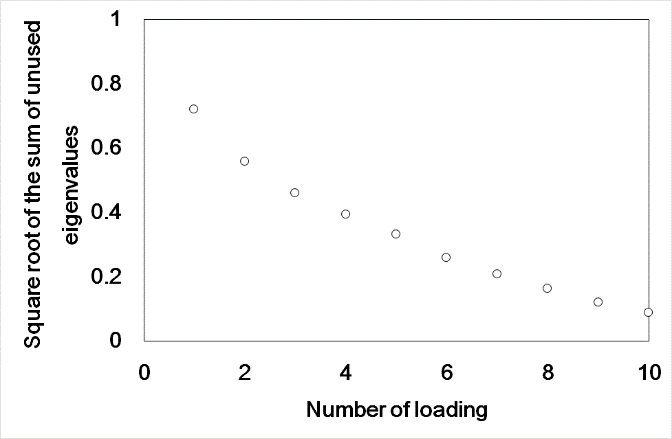

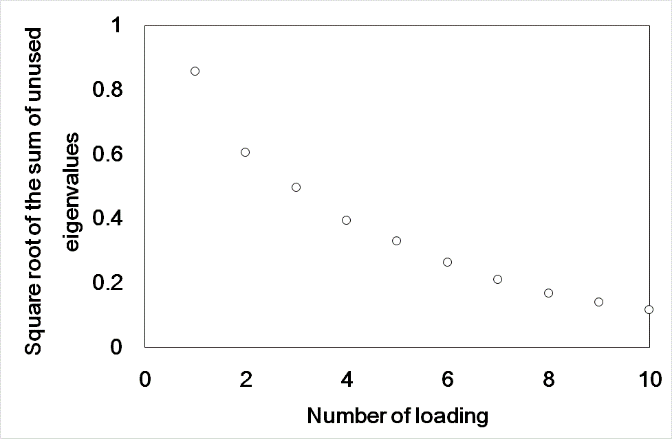


**(*a*)**

**(*b*)**

**Figure S-4.** Dependence of square root of the sum of unused eigenvalues as a function of number of loading (scree plot) for (a) Hamilton and (b) Simcoe.
